# The association of biomarkers with pain and function in acute and subacute low back pain: a secondary analysis of an RCT

**DOI:** 10.1186/s12891-022-06027-9

**Published:** 2022-12-05

**Authors:** Valerio Tonelli Enrico, Michael Schneider, Mitchell Haas, Nam Vo, Wan Huang, Christine McFarland, Nick Weber, Gwendolyn Sowa

**Affiliations:** 1 Ferguson Laboratory for Orthopaedic and Spine Research, Department of Orthopaedic Surgery, University of Pittsburgh, 200 Lothrop Street, Room E1612, BST, Pittsburgh, PA 15261 USA; 2 Department of Physical Therapy, University of Pittsburgh, 100 Technology Dr, Pittsburgh, PA 15219 USA; 3 Clinical and Translational Science Institute, University of Pittsburgh, Forbes Tower, Suite 7057, Pittsburgh, PA 15213 USA; 4 Integrative Health & Wellbeing Program, University of Minnesota, MMC 505; 420 Delaware Street S.E, Minneapolis, MN 55455 USA; 5 Department of Physical Medicine and Rehabilitation, School of Medicine, University of Pittsburgh, Kaufmann Medical Building, Suite 910; 3471 Fifth Avenue, Pittsburgh, PA 15213 USA; 6 Shirley Ryan AbilityLab, Chicago, 355 E Erie St, Chicago, IL 60611 USA; 7 Department of Physical Medicine & Rehabilitation, Northwestern University Feinberg School of Medicine, 710 N Lake Shore Dr #1022, Chicago, IL 60611 USA

**Keywords:** Acute low back pain, Biomarkers, Chiropractic, Medical care, Inflammation, Spinal manipulation

## Abstract

**Background:**

Low back pain (LBP) is a common musculoskeletal condition and a major cause of disability worldwide. Previous studies have found associations of biomarkers with pain and pain-related disability in LBP patients. This study aimed to explore the association between serum biomarkers and pain and disability in patients with acute or subacute axial LBP.

**Methods:**

This study was ancillary to a parent randomized controlled trial. Enrolled participants were randomized into three intervention groups: one of two types of spinal manipulation or medical care. In the parent study, 107 adults who experienced a new episode of LBP within 3 months prior to enrollment were recruited. For this study, 90 of these 107 participants consented to have blood samples obtained, which were drawn immediately before the beginning of treatment. Seven biomarkers were chosen based on previous literature and analyzed. Clinical outcomes were pain and Oswestry Disability Index (ODI) evaluated at baseline and 4 weeks. Spearman’s |r| was used to study the association of initial levels of each biomarker with pain and ODI scores at baseline and with changes in outcome scores from baseline to 4 weeks (end of treatment) within each intervention group.

**Results:**

At baseline, 4 of 7 biomarkers had an association with pain that was |r| ≥ .20: neuropeptide Y (NPY) (*r* = 0.23, *p* = .028), E-Selectin (*r* = 0.22, *p* = .043), vitamin D ((*r* = − 0.32, *p* = .002), and c-reactive protein (CRP) (*r* = 0.37, *p* = .001). No baseline biomarker had an association with disability that was |r| ≥ 0.20. For the correlations of baseline biomarkers with 4-week change in outcomes, vitamin D showed a correlation with change in disability and/or pain (|r| ≥ 0.20, *p* > .05) in manipulation-related groups, while CRP, NPY, and E-selectin along with TNFα, Substance P and RANTES showed at least one correlation with change in pain or disability (|r| ≥ 0.20, p > .05) in at least one of the treatment groups.

**Conclusions:**

In 90 LBP patients, the analyzed biomarkers, especially vitamin D, represent a small set of potential candidates for further research aimed at individualizing patient care. Overall, the associations investigated in the current study are an initial step in identifying the direct mechanisms of LBP and predicting outcomes of manipulation-related treatments or medical care.

**Trial registration:**

ClinicalTrials.gov Identifier: NCT01211613, Date of Registration: September 29, 2010, https://clinicaltrials.gov/ct2/show/NCT01211613?term=schneider&cond=Low+Back+Pain&cntry=US&state=US%3APA&draw=2&rank=1

## Background

Low back pain (LBP) is one of the most common musculoskeletal conditions requiring medical care and contributing to patient impairment and disability. Over 25% of the general population reports LBP at any given time [1], with a lifetime incidence exceeding 80% and a lifetime prevalence of around 40% [2]. However, the care of LBP represents one of the most significant challenges facing musculoskeletal care today. In the US, estimated annual costs for LBP alone were over $85 billion in 2005 [3], while in 2016, the combined cost for neck and LBP accounted for over $134 billion [4], highlighting the significant spending to treat this condition.

Despite these high costs, treatment outcomes and rates of disability have not improved [5], which may partly explain the growing interest in complementary medical treatments. Data from the 2012 National Health Interview Survey, Alternative Health Supplement demonstrate that 41.2% of the LBP population used complementary and alternative medicine approaches for pain relief, with herbal supplements, massage, and chiropractic manipulation being the most common therapies [6]. Previous data demonstrate short-term reductions in self-reported disability and pain scores in patients treated with manual thrust manipulation compared to usual medical care [7].

Diagnostic tools are necessary to identify patients with LBP who are likely to respond to different treatments, including manual therapy. Currently, a reliable method for selecting the most appropriate care for LBP patients is unavailable; identifying specific subgroups of patients would most likely improve outcomes and make interventions more cost-effective [8]. Biomarkers have the potential to become a valuable and novel tool in establishing individualized treatment plans by providing information about the mechanisms of action of clinical treatments and their related outcomes.

Some biomarkers have already shown an association with pain and pain-related disability in LBP patients, superior to any contribution by imaging studies [9]. Biomarkers have also garnered attention in manipulative treatment in several prior small studies. In a recent systematic review and meta-analysis of randomized clinical trials (RCTs) [10], varying degrees of evidence showed significant associations between spinal manipulation and various biomarkers up to 2 hours after the intervention. Overall, non-pooled results from 8 studies showed moderate evidence that spinal manipulation was associated with short-term biochemical markers changes after receiving spinal manipulation compared to controls (325 participants total). In the three studies (*n* = 173) investigating the effect of spinal manipulation on inflammatory biomarkers, moderate-quality evidence was found that spinal manipulation is more impactful than the control in influencing the short-term concentrations of tumor necrosis factor-alpha (TNFα) and interleukin 1 and 2 (IL-1, IL-2). These studies are characterized by a short-term assessment period of biomarkers’ levels after spinal manipulation, and they did not assess associations with clinical outcomes.

A more recent systematic review that focused on the effect of spinal manipulation and markers of immune function [11] found that the evidence is too weak to draw definite conclusions on the effect of spinal manipulation on immune system outcomes. The review acknowledged that short-term changes in immunological markers exist after spinal manipulation; yet, the only study that included specific blood markers of inflammation was a 2006 study by Teodorczyk-Injeyan et al., which was also included in the Kovanur-Sampath et al. review from 2017 mentioned previously. The authors also concluded that the clinical implications of such findings were unclear.

A non-randomized clinical trial [12] showed that IL-6 and C-reactive protein (CRP) levels decreased towards the levels of the healthy, pain-free control group in a small study of 10 patients with chronic LBP treated with nine sessions of mechanical-assisted manipulation. However, this study did not include any control subjects with LBP. Moreover, a study comparing 10 subjects with chronic LBP to 10 non-LBP controls showed decreased circulating levels of 5-hydroxyindoleacetic acid and serotonin4 hours after osteopathic manipulative treatment [13]. A case report shows a reduction in TNFα levels after manipulative therapy in two cervicogenic headache patients [14] [12]..

Two systematic reviews were recently published on the relationship between inflammatory biomarkers and LBP. In one [15], the authors concluded that CRP, TNFs, and IL-6 were positively associated with non-specific LBP. In the other study [16], the authors found that CRP levels were associated with acute non-specific LBP and TNFαwith chronic non-specific LBP. These reviews did not look at clinical effects or changes specific to spinal manipulation treatments. Interestingly, a newly published study found that a cluster of subjects with acute LBP characterized by an early combined presence of both IL-6 and CRP was associated with better recovery at 12 months than a different group characterized by elevated TNFα and depressive symptoms. Even though this study was observational, the association between inflammatory markers and clinical outcomes points to a potential role for such markers in determining the trajectory of recovery for individuals with acute LBP.

Overall, as pointed out in the most recent systematic reviews [15, 16], the current literature is characterized by numerous limitations that affect study quality, such as small sample size and lack of proper control groups. Two others are particularly relevant to our work. The first is not examining the changes in biomarker levels outside a narrow time window immediately before and after the intervention. The second limitation is focusing only on changes in biomarker levels without correlating these changes with clinically relevant outcomes such as reducing pain intensity or physical function. These limitations leave potentially relevant questions unanswered about the spinal manipulation’s medium- to long-term impact on homeostasis, physiological variances, and clinically meaningful outcomes. The current study aimed to explore the association of serum biomarkers with baseline and changes in self-reported pain and disability in LBP patients treated with spinal manipulation or standard medical care for acute and subacute axial LBP.

## Methods

### Recruitment

This biomarkers study was ancillary to a parent RCT that investigated patient outcomes in response to two types of spinal manipulation and medical care [7]. In the parent RCT, 107 adults (18 years or older) who experienced a new episode of LBP within the 3 months prior to enrollment were recruited from a single academic center. The study was conducted between November 2010 and March 2013, and was designed to adhere to CONSORT guidelines. Eligible subjects (Table 1) had an Oswestry Disability Index score between 20 and 70 points (0–100 scale) and a numeric pain rating score between 3 and 8 points (0–10 scale). Patients were excluded if they had: 1) prior history of lumbar spine surgery, unstable spondylolisthesis, spinal stenosis, or scoliosis > 20°; 2) signs or symptoms suggestive of nerve root tension or neurological deficit in the lower extremity; 3) red flag findings (including a history of metastatic cancer, osteoporosis, long-term corticosteroid use, unexplained weight loss of > 10% of body weight, spinal pain associated with fever, and severe night pain unrelieved by medication); 4) were receiving any physical therapy, chiropractic therapy, or any other manual therapy for this episode of LBP (within the past 3 months) or any on-going medical care for this episode of LBP; or 5) had a current use of opiate or other prescription medications for LBP, or were pregnant.

**Table 1:** Inclusion and exclusion criteria

Inclusion criteria	Exclusion criteria
Participants were required to have a new LBP episode within the previous 3 months.	Chronic LBP (> 3 months duration)
At least 18 years of age	Previous chiropractic, medical, or physical therapy treatment for the current LBP episode
Speak/understand English	Radicular features including leg pain distal to the knee, numbness/weakness of the lower leg, or positive nerve root tension/neurological signs
Minimum levels of self-reported pain (3 on 0–10 scale)	Current use of prescription pain medications
Minimum levels of self-reported disability (20 on 0–100 scale)	Contraindications to spinal manipulation, including: • Previous history of metastatic cancer • Severe osteoporosis • Fracture or instability • Prolonged anticoagulant or oral steroid use
Agree to be randomized	
Agree to attend 2 office visits per week for 4 weeks	
Agree to cooperate with follow-up data collection	
Agree to have blood drawn	

Individuals were randomized into one of three intervention groups: *manual-thrust manipulation*, using high-velocity, low-amplitude thrust manipulation using standard chiropractic methods by a licensed chiropractor (group 1); *mechanically-assisted manipulation* using an Activator IV Instrument (Activator Methods International Ltd., Phoenix, AZ) (group 2); or *standard medical care* provided by a board-certified physician in physical medicine and rehabilitation (group 3) [7]. The high-velocity, low-amplitude thrust manipulation varied from patient to patient but was either side posture, diversified, or drop table technique. Furthermore, the location of the adjustment (manipulation technique) varied from patient to patient, based upon their individual needs. The clinicians were allowed to adjust the sacroiliac joints and/or any facet joints from L5-S1 up to T6–7. The protocol did not allow for any manipulation of the cervical or upper thoracic segments or any extremity joints. Moreover, all patients assigned to manipulation were seen twice a week for 4 weeks. The standard medical care approach consisted of a protocol in line with guidelines for the clinical management of non-specific LBP [17]. Randomization was performed using a rank-based adaptive allocation approach; more information on the methodology can be found in the published manuscript from the parent study [7]. Blinding was not possible due to the study design.

Before randomization, subjects were asked if they were willing to participate in the ancillary biomarkers study, which would require them to have blood drawn upon initiating the course of treatment. Subjects who agreed to participate in the ancillary study were taken through a separate consent process, and a separate University of Pittsburgh Institutional Review Board approval was obtained for the biomarker study. Participation in the biomarker study did not influence participation in the parent RCT. Of the 107 adults with LBP who participated in the parent RCT, 90 agreed to participate in the ancillary biomarker study (group 1, *n* = 33; group 2, *n* = 27; group 3, *n* = 30). For the analysis of the 4-week change in pain and disability scores, 4 subjects were lost to follow-up, one data was missing for the change in pain variable, and two data were missing for change in Oswestry Disability Index (ODI) (group 1, *n* = 30; group 2, *n* = 26; group 3, *n* = 29 for pain and *n* = 28 for ODI). No other subjects were excluded for any other reasons.

No power calculation was performed for this secondary analysis since a convenience sample was used. All investigators involved in the biomarker analysis remained blinded to treatment assignment until after the completion of the trial.

### Blood sampling and assays

Blood samples were drawn from participants immediately before the first treatment session at baseline. About 10 ml of venous blood was collected into Serum Separation Tubes Vacutainers (4 ml, Ref# 367977) and EDTA pre-coated vacutainers (6 ml, Ref# 367863), both from BD (Fullerton, CA) and kept in ice. Serum Separation Tubes Vacutainers were immediately sent to the nearby clinical lab to measure Vitamin D and CRP, whereas the samples in the EDTA vacutainers were centrifuged within 2 hours at 1000 g for 10 min for enzyme-linked immunosorbent assay (ELISA). The resulting plasma was pipetted out and divided into Eppendorf tubes, which were pre-labeled with patients ID, and immediately frozen at -80oC. Plasma samples were analyzed by ELISA using a commercially available 96-well ELISA kits: Neuropeptide Y (NPY) and E-selectin from (RayBiotech Inc., Norcross, GA), and Substance P, TNF-a, and RANTES (regulated on activation, normal T cell expressed and secreted, also known as C-C Motif Chemokine Ligand 5 (CCL5)) from Cayman Chemical (Ann Arbor, MI). All assays were performed in duplicate and following the manufacturer’s instructions, and the intraassay and interassay coefficients of variation are < 10% for all ELISAs. The selection of biomarkers was based on previous reports of their association with LBP [9, 18–22]. Vitamin D and CRP levels were also obtained from the samples taken immediately before treatment; a local clinical lab performed their standard analysis.

### Clinical data collection

Baseline demographic and clinical information, including age, BMI, gender, race, employment status, general health, presence/absence of metabolic syndrome, tobacco use, fear-avoidance beliefs [23], baseline NSAID use, treatment expectancy as measured by the Treatment Credibility Expectancy Questionnaire and intermittent vs. constant nature of symptoms were collected. Primary clinical outcomes were pain measured by the von Korff pain scale [24] and disability measured by the Oswestry Disability Index (ODI) [25]. Pain and ODI scores were measured at the beginning of the first (baseline) and last (4-weeks) treatment sessions.

### Statistical analysis

The strength of the relationship between the pain/disability variables and each of the biomarkers was quantified using non-parametric Spearman’s rank-order correlation (Spearman’s rho [r]). Baseline correlations were computed using all 90 subjects because the data from the parent RCT were collected prior to randomization and interventions.

The correlations of improvement of pain and disability outcomes with baseline biomarkers were evaluated by treatment type. This approach was chosen because of the effects of the interventions on the outcomes [7]. Scatterplots were visually inspected for apparent violation of the assumption of a monotonically increasing or decreasing association between variables. None were noted.

## Results

The majority of participants from the randomized trial agreed to join the biomarker study (90/107 = 84%) (Table 2). A chi-square test showed no significant difference between the study groups in the proportion of participants from the parent study who enrolled (*p* = .358): group 1 = 33/35 (85%), group 2 = 27/37 (89%), and group 3 = 30/35 (77%).

**Table 2:** Baseline demographics

		All participants	Group 1	Group 2	Group 3
Age		38.7 (14.1)	37.7 (14.7)	36.6 (14.5)	39.7(11.8)
BMI		29.2 (6.9)	29.3 (7.6)	31.3 (6.7)	27.5 (6.2)
Sex	F	*n* = 55 (61%)	*n* = 21 (64%)	*n* = 16 (60%)	*n* = 18 (60%)
	M	*n* = 35 (39%)	*n* = 12 (36%)	*n* = 11 (40%)	*n* = 12 (40%)
Race	White	*n* = 56 (63%)	*n* = 22 (66%)	*n* = 16 (59%)	*n* = 18 (60%)
	Black	*n* = 25 (27%)	*n* = 7 (21%)	*n* = 8 (30%)	*n* = 10 (33%)
	Asian	*n* = 3 (3%)	*n* = 1 (3%)	*n* = 0 (0%)	*n* = 2 (6%)
	Mixed race	*n* = 6 (7%)	*n* = 3 (10%)	*n* = 3 (11%)	*n* = 0 (0%)
Smoking status	Never	*n* = 38 (42%)	*n* = 13 (39%)	*n* = 9 (33%)	*n* = 16 (53%)
	Currently	*n* = 20 (22%)	*n* = 11 (34%)	*n* = 5 (19%)	*n* = 4 (14%)
	Past	*n* = 32 (36%)	*n* = 9 (27%)	*n* = 13 (48%)	*n* = 10 (33%)
Exercise	No (1)	*n* = 6 (7%)	*n* = 4 (12%)	*n* = 0 (0%)	*n* = 2 (7%)
	No (2)	*n* = 16 (18%)	*n* = 6 (18%)	*n* = 5 (18%)	*n* = 5 (17%)
	No (3)	*n* = 14 (16%)	*n* = 5 (15%)	*n* = 5 (18%)	*n* = 4 (13%)
	Yes (4)	*n* = 18 (20%)	*n* = 6 (18%)	*n* = 8 (30%)	*n* = 4 (13%)
	Yes (5)	*n* = 35 (39%)	*n* = 11 (33%)	*n* = 9 (34%)	*n* = 15 (50%)
	Missing	*n* = 1 (1%)	*n* = 1 (3.3%)	*n* = 0 (0%)	*n* = 0 (0%)
General Health	Poor	*n* = 0 (0%)	*n* = 0 (0%)	*n* = 0 (0%)	*n* = 0 (0%)
	Fair	*n* = 6 (7%)	*n* = 2 (6%)	*n* = 2 (7%)	*n* = 2 (6%)
	Good	*n* = 40 (44%)	*n* = 13 (39%)	*n* = 13 (48%)	*n* = 14 (47%)
	Very good	*n* = 37 (41%)	*n* = 17 (48%)	*n* = 10 (37%)	*n* = 11 (37%)
	Excellent	*n* = 6 (7%)	*n* = 1 (3%)	*n* = 2 (7%)	*n* = 3 (10%)
	Missing	*n* = 1 (1%)	*n* = 1 (3%)	*n* = 0 (0%)	*n* = 0 (0%)
Pain Score		5.7 (1.4)	5.5 (1.4)	6.1 (1.4)	5.6 (1.4)
ODI		34.3 (9.3)	33.8 (9.6)	35.0 (10.2)	33.9 (8.5)

All participants (*n* = 90); Group 1 (*n* = 33) = manual manipulation; Group 2 (*n* = 27) = mechanical manipulation; Group 3 (*n* = 30) = medical care

(1)= no intention to in the next 6 months; (2)= intention to in the next 6 months; (3)= intention to in the next 30 days; (4)= have been for LESS than 6 months; (5)= have been for MORE than 6 months

For Age, BMI, Pain score, and ODI, mean and (Standard Deviation) is reported

For Sex, Race, Smoking Status, Exercise and General Health, number of participants (n) and percentage within that category (%) is reported

In the baseline analysis of all 90 subjects as a whole (Table 3), an association of r ≥ 0.20 was found for pain with NPY(*r* = 0.23, *p* = .028) and E-Selectin (*r* = 0.22, *p* = .043). Additionally, an association of |r| ≥ 0.30 was found for pain with vitamin D (*r* = − 0.32, *p* = .002) and CRP (*r* = 0.37, *p* = .001). At baseline, there were no correlations of the 7 biomarkers with disability at the level of |r| ≥ 0.20.

**Table 3:** Correlation between baseline biomarker levels and baseline pain/disability scores (*n* = 90)

Biomarker	Pain		ODI	
	r	*p*	r	*p*
NPY	**.23**	**.028**	.09	.375
E-Selectin	**.22**	**.043**	.17	.108
TNFα	.09	.426	.16	.124
SubP	.17	.123	.02	.876
RANTES	−.01	.968	−.09	.422
vitamin D	**−.32**	**.002**	−.16	.137
CRP	**.37**	**.000**	.16	.122

*NPY* Neuropeptide Y, *TNFα* Tumor necrosis factor-alpha, *SubP* substance P, *RANTES* Regulated upon activation, normal t-cell expressed and presumably secreted, *CRP* c-reactive protein, *r* Spearman’s correlation coefficient, *ODI* Oswestry Disability Index score

The associations between baseline biomarkers levels and changes in pain from baseline to 4 weeks by treatment group are presented in Table 4. Ten correlations of biomarkers with disability improvement and 6 with pain improvement had a magnitude of |r| ≥ 0.20 (*p* > .05). Of these, 4 biomarkers had correlations with disability improvement that reached |r| ≥ 0.30 (p > .05).

**Table 4:** Correlations between baseline biomarker levels and 4- week change in pain/disability scores

Biomarker	Treatment	Change in pain		Change in ODI	
		r	p	r	p
NPY	Group 1	−.15	.430	−.13	.480
	Group 2	.19	.347	−.03	.857
	Group 3	−.29	.129	−.24	.202
E-Selectin	Group 1	.04	.834	−.00	.978
	Group 2	.10	.615	.38	.056
	Group 3	.07	.711	.08	.674
TNFα	Group 1	.16	.396	.28	.124
	Group 2	−.15	.449	.13	.516
	Group 3	.06	.741	−.18	.357
SubP	Group 1	−.00	.982	−.14	.436
	Group 2	.10	.612	.33	.095
	Group 3	.13	.494	.28	.186
RANTES	Group 1	.24	.197	−.04	.846
	Group 2	−.16	.434	−.01	.946
	Group 3	−.05	.783	−.26	.186
Vitamin D	Group 1	.24	.204	.33	.070
	Group 2	−.05	.812	−.24	.231
	Group 3	−.15	.443	−.02	.923
CRP	Group 1	−.26	.165	−.24	.199
	Group 2	.20	.311	.31	.111
	Group 3	.26	.179	.06	.748

Group 1 (*n* = 33) = manual manipulation Group 2 (*n* = 27) = mechanical manipulation Group 3 (*n* = 30 for pain; *n* = 29 for ODI) = medical care

*NPY* Neuropeptide Y, *TNFα* Tumor necrosis factor-alpha, *SubP* Substance P, *RANTES* Regulated upon activation, normal t-cell expressed and presumably secreted; D, *CRP* c-reactive protein, r Spearman’s correlation coefficient, *ODI* Oswestry Disability Index

## Discussion

This study was the first to examine molecular biomarkers and clinical metrics in patients with axial LBP randomized to manipulation versus standard medical care. As a secondary analysis of an RCT dataset, the goal was to examine and identify potential biomarkers of interest related to pain and functional disability for future validation.

The initial CRP levels were correlated with baseline pain values, with a |r| value of 0.37.; however, the correlation between CRP and baseline function showed a much smaller correlation. CRP also showed correlation coefficients of magnitude between |*r*| = 0.20 and |*r*| = 0.31 across the 3 treatment groups associated with changes in pain and in the manual manipulation and mechanical manipulation groups associated with changes in ODI. Previous literature has shown an association between CRP and LBP [15, 16, 26]. Interestingly, higher CRP levels in the acute phase have been associated with better recovery than initial lower CRP levels (when lower levels are associated with other comorbidities, such as depression or sleep disturbances) [26]; similar findings were replicated in the 12-months outcomes of a recent prospective cohort [27]. Our findings partially align with previous results and may eventually translate into clinical practice [28].

The current lack of understanding of the mechanisms by which these markers affect pain and treatment trajectory underscores the need for studying this potential marker in future studies. This could potentially provide further insight into the interaction between CRP levels and LBP and potential mechanisms of action of manipulation. Nonetheless, this result highlights the need to consider this biomarker for future research on its mechanistic role in spinal manipulation.

The second important finding was that baseline Vitamin D concentrations were inversely correlated with baseline pain (*r* = − 0.32). This baseline correlation is in line with growing evidence on the contributing role of vitamin D on musculoskeletal pain and, more specifically, on LBP [29]. Even though the evidence is not conclusive, there is growing consensus that decreased levels of vitamin D are associated with pain syndromes [30] and that vitamin D could be administered to people to help decrease pain [31], especially if their initial vitamin D levels are low [32]. Vitamin D levels were also correlated with pain changes and ODI changes in the manual manipulation group and with changes in ODI in the mechanical manipulation group, with coefficients ranging between |*r*| = 0.24 and |*r*| = 0.31.

The evidence from the current study suggests that vitamin D could be a potential predictive biomarker for physical treatment outcomes. Besides its effects on the skeletal system, vitamin D also influences nervous, immune, and cardiovascular systems through its vital role in calcium metabolism. Therefore, the explanation for the possible correlation between baseline vitamin D with the manipulation outcomes could be multifactorial and prompts further investigations. Unfortunately, and to the best of our knowledge, no research on this topic has been performed yet. However, a review article has pointed out that vitamin D presurgical levels could predict surgical outcomes [33]. Though manipulation is not an intervention as invasive as surgery, the outcomes of manipulation may similarly be impacted by the baseline vitamin D levels. We suggest that vitamin D should be included in future studies on pain, biomarkers, and treatment outcomes, with particular attention to manipulation treatment to investigate this relationship further.

Both CRP and vitamin D had a Spearman’s Rho above 0.30; while this value is generally acknowledged in research as low to moderate strength, further discussion is warranted within the context of biomarkers and LBP. Regarding biomarkers’ association with clinical features, previous literature has been published with a similar correlation coefficient when looking at clinical variables such as pain and change in functional score (also assessed with ODI) [9, 34]. It could be that the strength of correlation of a single blood marker should be considered within the context of how it correlates with clinical features and their application in the decision-making process for clinical treatment. In addition, it is implausible that any single biomarker alone would be able to determine the clinical course of action, but rather should be considered in the context of a complex clinical presentation. In this light, even a 0.20 correlation may contribute to making impactful decisions regarding clinically complex patients. This is particularly true when considering that the approach often used for clinical decision-making for LBP treatment relies heavily on diagnostic imaging, which has not been found to be correlated with pain and function [9, 35, 36]. While these associations are not sufficient independently to impact clinical guidelines, they represent an important contribution to potentially supplementing diagnostic and prognostic tools.

Furthermore, we found associations between baseline levels of NPY and E-Selectin, both of which had a correlation coefficient above 0.20. NPY levels have been found in previous literature to be correlated with chronic pain [37], though our finding in acute and subacute LBP subjects is novel, warranting further research involving this biomarker. The literature on E-Selectin and pain shows an unclear trend. There is evidence of a significant correlation between baseline E-Selectin and pain levels for patients with chronic LBP (but not acute) [19]. However, another study found no association between E-selectin levels and pain or pain-related function in an older adult cohort with disc degeneration [9]. Our study highlights that pain levels in acute and subacute LBP patients may be associated with heightened levels of E-selectin, thus adding evidence to an area of research that needs clarification [37–40].

This study allowed us to explore the potential association between biomarkers and treatment outcomes. This topic has been gaining increasing attention in the literature, having been explored across this population and by treatment groups. The examined biomarkers represent only a sampling of potential biomarkers that may show important associations. We chose to study well-known inflammatory and pain biomarkers in this initial effort to identify candidate predictive biomarkers. While clearly, additional validation and an examination of a broader array of biomarkers are needed, these findings support future research on the potential utility of circulating biomarkers in this population.

Considering the small numbers of patients in each treatment group, though no significant correlations were found, even a 0.20 correlation may contribute to making impactful decisions when choosing manipulation-related treatments over usual medical care for LBP. Interestingly, vitamin D showed a change in disability and/or change in pain (|r| ≥ 0.20) in manipulation-related treatment groups only. This pattern warrants further study with a larger study population. The correlation coefficient of the other analyzed biomarkers was at least 0.20 associated with pain and/or ODI changes at 4 weeks in one of three treatment groups. These results are difficult to interpret since they present across different treatment groups.

The small sample sizes for each group are clear limitations to these results. These limitations are even more relevant because we used this exploratory analysis for multiple comparisons. However, this limitation is tempered by the need for novel targets and the risk of missed findings. This study had some other limitations, including its small sample size and exploratory nature. The data distribution could not be considered normal, which was addressed using non-parametric statistical analysis. Furthermore, the average BMI value for the included population was elevated (29 ± 5.9), which has potential implications for the inflammatory markers. Nonetheless, numerous studies have presented similar average BMI values [38–41], which point to the complexity of controlling for such a variable when older age and higher BMI are closely confounded.

Another limitation of this study was the absence of a control group that received no treatment, which would have allowed for observation of changes in biomarker levels over time via natural history. This improvement could have been possible by having participants assigned to a wait-list control group and would have added a layer of information in comparing the various interventions. Additional work should be pursued in this area to gain further insight into the role of biomarkers and changes in clinical outcomes. Another limitation was the lack of a blood draw immediately after the first intervention session; this would have made the study more comparable to previous research in this article’s literature review section and could help form future research questions. However, on the contrary, the inclusion of later time points for clinical outcomes is a strength of this study since they support the association between baseline biomarkers and clinical outcomes. Last, confounders could have impacted the analysis results, such as medications, supplements, and other conditions which could contribute to the biomarker pool, even though the RCT design is generally accepted to address this potential issue.

In identifying relevant serum biomarkers, previous work suggests that panels of biomarkers may provide greater predictive power than any single biomarker alone [9]. Also, combining molecular biomarkers with clinical metrics may likely prove to be the most helpful approach in identifying those patients most expected to benefit from a given treatment. This combined approach represents a potential new avenue for sub-classifying patients and developing individualized treatment plans to address sub-group differences. Overall, the associations we observed do not point at robust, direct mechanisms but likely depend on a more comprehensive set of physiological and clinical interactions that are still not understood. More research is thus warranted in this area.

## Conclusion

We found small correlations of vitamin D, CRP, NPY, and E-selectin with pain and disability at baseline and weak correlations of baseline vitamin D with improvement in outcomes following a four-week course of treatment with manipulative-related therapies. For acute and subacute LBP. We believe these data make an important contribution to the field of research on biomarkers and pain, suggesting that these serum biomarkers could have a potential role in further study of the mechanisms of prevalent LBP and prediction in related treatment outcomes and therefore warrant further evaluation. Future biomarkers research may help inform a personalized medicine approach to LBP management.

## Data Availability

The datasets used and/or analyzed during the current study are available from the corresponding author on reasonable request and with a properly executed data use agreement.

## Abbreviations

NPY: Neuropeptide Y
TNFα: Tumor necrosis factor-alpha
SubP: Substance P
RANTES: Regulated upon activation, normal t-cell expressed and presumably secreted
CRP: C-reactive protein
ODI: Oswestry Disability Index
LBP: Low back Pain
RCT: Randomized clinical trial
BMI: Body Mass Index
EDTA: Ethylene-diamine-tetraacetic acid
CCL5: C-C Motif Chemokine Ligand 5
